# Identification of miRNAs Differentially Expressed in Human Epilepsy with or without Granule Cell Pathology

**DOI:** 10.1371/journal.pone.0105521

**Published:** 2014-08-22

**Authors:** Silvia Zucchini, Gianluca Marucci, Beatrice Paradiso, Giovanni Lanza, Paolo Roncon, Pierangelo Cifelli, Manuela Ferracin, Marco Giulioni, Roberto Michelucci, Guido Rubboli, Michele Simonato

**Affiliations:** 1 Department of Medical Sciences, Section of Pharmacology and Neuroscience Center, University of Ferrara, Ferrara, Italy; 2 National Institute of Neuroscience, Torino, Italy; 3 Laboratory for Technologies of Advanced Therapies (LTTA), University of Ferrara, Ferrara, Italy; 4 Department of Biomedical and NeuroMotor Sciences (DiBiNeM), Section of Pathology, Bellaria Hospital, Bologna, Italy; 5 Department of Morphology, Surgery and Experimental Medicine, Section of Pathology, University of Ferrara, Ferrara, Italy; 6 Ri.MED Foundation, Palermo, Italy; 7 Department of Morphology, Surgery and Experimental Medicine, Section of Pathology, Oncology and Experimental Biology, University of Ferrara, Ferrara, Italy; 8 IRCCS Institute of Neurological Sciences, Section of Neurosurgery, Bellaria Hospital, Bologna, Italy; 9 IRCCS Institute of Neurological Sciences, Section of Neurology, Bellaria Hospital, Bologna, Italy; 10 Danish Epilepsy Center, Epilepsihospital, Dianalund, Denmark; University of Modena and Reggio Emilia, Italy

## Abstract

The microRNAs (miRNAs) are small size non-coding RNAs that regulate expression of target mRNAs at post-transcriptional level. miRNAs differentially expressed under pathological conditions may help identifying mechanisms underlying the disease and may represent biomarkers with prognostic value. However, this kind of studies are difficult in the brain because of the cellular heterogeneity of the tissue and of the limited access to fresh tissue. Here, we focused on a pathology affecting specific cells in a subpopulation of epileptic brains (hippocampal granule cells), an approach that bypasses the above problems. All patients underwent surgery for intractable temporal lobe epilepsy and had hippocampal sclerosis associated with no granule cell pathology in half of the cases and with type-2 granule cell pathology (granule cell layer dispersion or bilamination) in the other half. The expression of more than 1000 miRNAs was examined in the laser-microdissected dentate granule cell layer. Twelve miRNAs were differentially expressed in the two groups. One of these, miR487a, was confirmed to be expressed at highly differential levels in an extended cohort of patients, using RT-qPCR. Bioinformatics searches and RT-qPCR verification identified ANTXR1 as a possible target of miR487a. ANTXR1 may be directly implicated in granule cell dispersion because it is an adhesion molecule that favors cell spreading. Thus, miR487a could be the first identified element of a miRNA signature that may be useful for prognostic evaluation of post-surgical epilepsy and may drive mechanistic studies leading to the identification of therapeutic targets.

## Introduction

The microRNAs (miRNAs) are small size endogenous non-coding RNAs that regulate the expression of target mRNAs at post-transcriptional level [1]. To date, more than 1000 human miRNAs have been identified, about 50% of which are expressed in the brain. miRNAs have been demonstrated to be involved in several brain functions, many of which may be implicated in epilepsy and epileptogenesis, like cell death, neurogenesis, synaptic plasticity [2],[3]. Indeed, silencing miR-134 using a specific antagomir exerted prolonged seizure-suppressant and neuroprotective actions in a murine model [4]. Thus, understanding which specific miRNAs are differentially expressed in epilepsy may help to identify the mechanisms underlying the disease. Moreover, differentially expressed miRNAs may represent biomarkers that identify specific subpopulations of epileptic patients, holding a prognostic value [5].

Microarray platforms allow screening and identifying miRNAs differentially expressed under pathological conditions. Experimental studies have profiled miRNA expression in animal models of epilepsy [6],[7],[8],[9] and profiling studies have been also recently published using hippocampi resected from temporal lobe epilepsy (TLE) patients [10],[11]. However, some outstanding obstacles make difficult the interpretation of data from microarray analysis of human brain samples. First, in most studies tissue is derived from autopsies or, potentially even worse, pathological tissue is from surgery samples and control tissue from autopsies. Post-mortem modifications are very likely to dramatically alter the molecular composition of the tissue, making the results questionable. Second, each brain area has a specific and complex cellular composition that changes (often markedly) in the course of diseases. Again, this makes interpretation of molecular data very difficult, because analysis of heterogeneous tissue homogenates does not allow identification of the cells where changes occur and because up-regulation of a molecule in one cell population may be obscured by down-regulation in another cell population.

One approach to overcome these problems is focusing on a well-defined cell population. For example, we focused here on a TLE-associated pathology of the granule cells of the hippocampus. Drug-resistant TLE is the most common type of epilepsy requiring surgical treatment, with a favorable postsurgical outcome in 60-70% of the patients. Based on the underlying etiology, TLE subtypes with different surgical prognosis have been described. Neuropathological classifications of epileptogenic lesions, including focal cortical dysplasias (FCD) [12], hippocampal sclerosis (HS) [13] and granule cell pathology (GCP) [14], define histopathological features and subtypes, allowing attempts to correlate clinical and pathological findings. Correlations with molecular markers, however, are still unavailable.

All patients included in this study underwent surgery for pharmacoresistant TLE and had HS type 1 [13]. All were similar for age, gender, clinical features of the disease. The most relevant difference was that half of the patients had no granule cell pathology (no GCP), whereas the other half had granule cell dispersion or bilamination (GCP type 2) [14], i.e. the single differential pathological feature was in a specific, isolable cell population. Therefore, the granule cell layer was laser-microdissected from all samples, total RNA was extracted from dissected tissues and the miRNAome profile was obtained using a miRNA microarray.

## Materials and Methods

### Patients

This study was approved by the Ethics Committee of Bologna (full name: *Comitato Etico Indipendente dell'Azienda USL della Città di Bologna*). A comprehensive written informed consent (also approved by the Ethics Committee of Bologna) was signed for the surgical treatment that produced the tissue samples, the related diagnostic procedures and the research use. All information regarding the human material used in this study was managed using anonymous numerical codes and samples were handled in compliance with the Helsinki declaration (http://www.wma.net/en/30publications/10policies/b3/).

Fourteen drug-resistant TLE patients candidate to epilepsy surgery were collected at the Epilepsy Surgery Center of the IRCCS Institute of Neurological Sciences of Bologna. All patients underwent detailed epileptological evaluation and wakefulness/sleep EEG. All patients also underwent continuous (24 hours) long-term video-EEG monitoring for seizure recording. Analysis of ictal clinical and EEG semiology and electroclinical correlations aimed to identify the epileptogenic area were performed.

Three Tesla MRI, and brain CT scan when necessary, were carried out. Electroclinical, neuroimaging, and neuropsychological data were discussed by the Epilepsy Surgery Team (epileptologists, neuroradiologists, neuropsychologists, neurosurgeons) to establish the site of the epileptogenic area and the surgical strategy.

### Surgery

All patients underwent tailored temporal lobe resection to remove the epileptogenic area, according to the data obtained during pre-surgical investigation. Essentially, surgery consisted of removing the temporal pole, the anterior neocortical lateral cortex, the uncus–entorhinal area and the hippocampus and parahippocampal gyrus. The main surgical specimens (hippocampus and/or temporal pole) were removed “en bloc” and spatially oriented to allow a proper histopathological examination.

### Histology and microdissection

Specimens were formalin fixed and paraffin embedded. They were de-waxed using Bio-Clear (Bio-Optica, Milan, Italy), washed in ethanol and stained with hematoxylin and eosin for histological diagnosis. Neuropathological evaluation was performed using the most recent classifications of HS, GCP and FCD [12],[13],[14], applying the recommended histochemical and immunohistochemical stains. Specimens either displayed no GCP or GCP type 2. Four different types of neuropathological features can define GCP type 2: 1) dispersion: rows of granule cells spread into the molecular layer and the distance between granule cells is increased; 2) ectopic granule cells: single ectopic granule cells are dispersed into the molecular layer; 3) clusters: ectopic granule cells form clusters within the molecular layer; 4) bilaminar: two granule cell layers, separated by a cell-free gap [14]. While patterns of granule cell loss (thinning and/or cell free gaps, GCP 1) occur isolated, patterns of architectural abnormalities (GCP 2) can came along with cell loss. Therefore, only sections in which no cell loss was detected (based on NeuN staining) where included in analysis.

**Figure 1:**
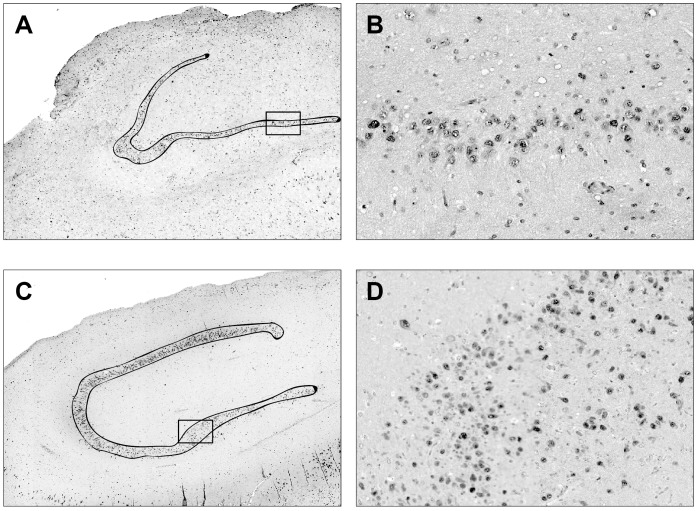
Laser microdissection of the human granule cell layer. Neu-N stained hippocampal sections prepared form a patient without granule cell pathology (A) and a patient with granule cell dispersion, i.e. type 2 granule cell pathology (C). The dissection line is in black. (B) and (D) are higher magnifications of the boxes in (A) and (C), respectively.

Ten-micron-thick sections were cut using a microtome and the dentate granule layer of the dentate gyrus was laser-dissected (**Fig. 1**) using the SL microcut microtest dissector (Nikon, Tokyo, Japan). Microdissected cells were captured in microcut transfer film (Nikon). Granule cells were collected in this manner from at least 3–4 slices per patient, in order to obtain an adequate quantity of RNA. Material from all sections of the same patient was pooled together, and total RNA extracted using an RNA purification kit (RecoverAll Total Nucleic Acid Isolation Kit, Ambion Life Technologies, CA, USA). Approximately 1.5 µg total RNA were obtained from each patient. Since miRNAs are more stable than mRNAs, they can be used for microarray analysis from formalin-fixed paraffin-embedded tissues [15]. We performed quality control checks on microarray hybridizations using the Agilent quality control (QC) tool in the Feature Extraction software. All samples passed the QC check.

### Microarray

Total RNA was used for microarray analysis (Human microRNA Microarray V3, #G4470C, Agilent Technologies, Santa Clara, CA, USA). This chip consists of 60-mer DNA probes and allows simultaneous analysis of almost 1200 human miRNA obtained from the Sanger miR-BASE database (Release 10.1). We employed approximately 100 ng total RNA per sample in each experiment. RNA labeling and hybridization were performed in accordance to manufacturer's indications. Agilent scanner and the Feature Extraction 10.5 software (Agilent Technologies) were used to obtain the microarray raw-data.

Microarray results were analyzed using the GeneSpring GX 12 software (Agilent Technologies). Data transformation was applied to set all the negative raw values at 1.0, followed by Quantile normalization and log2 transformation. Filters on gene expression were used to keep only the miRNAs detected in at least one sample (n = 536). The number of expressed miRNAs was 493 in the no GCP group, 464 in the GCP 2 group. Differentially expressed miRNAs were identified by comparing GCP type 2 vs. no GCP samples. A 2 fold-change filter (n = 141) and the unpaired t-test were applied (p<0.05; False Discovery Rate-FDR = 7%). Differentially expressed genes were employed in Cluster Analysis, using the Pearson correlation as a measure of similarity. For Cluster image generation, an additional step of normalization on gene median across all samples was added.

### miRNA qRT-PCR

Quantitative real-time PCR (qRT-PCR) analysis of hsa-miR-338-3p, 219-5p and 487a was performed using a TaqMan miRNA assay kit (Applied Biosystems) according to the manufacturer's instructions. Samples were run in triplicate at 95°C for 15 s and 60°C for 1 min using a CFX96 Touch Real-Time PCR Detection System (Applied Biosystems). Analysis was performed by the comparative threshold cycle (CT) method. rRNA U48 was used as reference gene. The relative amount of each miRNA in epileptic samples was calculated using the equation RQ  =  2^−CT^, where CT  =  (CT miRNA − CT U6 RNA). Similar results were obtained using rRNA U6 as reference gene.

### Bioinformatics

Target prediction was performed by comparative analysis of several databases, using an open-source database [16].

### ANTXR1 qRT-PCR

mRNA levels of *antrax receptor 1* (ANTXR1) (assay ID: Hs01120394) and *neuronal enolase 2* (ENO 2) (assay ID: Hs01102367), were determined using TaqMan Real-Time PCR, according to manufacturer's instructions (Applied Biosystems). Ten ng of total RNA were retro-transcribed using iScript Reverse Transcription Supermix (BIO-RAD). cDNA templates were amplified with TaqMan PreAmp Master Mix (Applied Biosystems), using pooled assay mix for ANTXR1 and ENO 2 and then assayed for gene expression as described previously. Each sample was analyzed in triplicate, in two independent experiments. The level of each mRNA was measured using Ct (threshold cycle) and the amount of target was calculated as described above for miRNAs. Gene expression levels were normalized using ENO 2 expression, as reported previously for this particular tissue [17].

### ANTXR1 immunohistochemistry

ANTXR1 immunostaining was performed by an automatic and clinically validated instrument based on Ventana Benchmark Ultra systems from Roche Tissue Diagnostics. This immunohistochemistry technique takes advantage of a new enhanced sensitivity biotin-free multimer technology system, based on direct linkers between peroxidase and secondary antibodies (ultraView Universal DAB Detection Kit, Ventana Medical System). The protocol provided for the automatic CC1 Ventana pre-treatment (Cell Conditioning Solution, Ventana) for 52 min, then the incubation for 2 hours with the anti-ANTXR1 antibody by titration (rabbit polyclonal, by ThermoFisher Scientific; 1: 100). Staining was visualized with the UltraView DAB procedure by Benchmark Ultra System. Sections were then counterstained with haematoxylin. Negative controls were treated identically except that the primary antibody was omitted. Sections of metastatic breast cancer were used as positive controls [18],[19]. Evaluation of data was performed by two expert neuropathologists (GM and BP) under double-blind conditions.

### Statistical analysis

For qRT-PCR data, comparisons between experimental groups were performed by using the Mann–Whitney U test. Differences between groups were considered significant when P < 0.05.

## Results

### Patients

Tissues from patients indicated in numbers in **Table 1** were employed for microarray analysis. Neuropathological examination evidenced that these patients had HS type 1 [13], which was associated with no granule cell pathology (no GCP) in 5 patients and with granule cell pathology (GCP type 2) in the other 5 [14]: GCP consisted of granule cell dispersion in 4 cases and bilaminar granule cell layer in one. The no GCP group was composed of 3 males and 2 females, with mean age at surgery of 44 (33–60), mean years after epilepsy diagnosis of 24 (7–38) and approximately 10 seizures per month before surgery (2 to >30); the GCP group was composed of 5 females, with mean age at surgery of 33 (31–37), mean years after epilepsy diagnosis of 23 (10–35) and approximately 10 seizures per month before surgery (3 to 15). An epileptogenic insult could be identified in only one of the no GCP cases (febrile convulsions), whereas all GCP cases had a history of febrile convulsions, one also a possible brain trauma (**Table 1**).

**Table 1:** Patients included in the study.

Patient number	Gender	Age at surgery	Epileptogenic insult	Years after diagnosis	Seizures per month	Drug therapy (current)	Pathology MTS [29]	Pathology MTS [30]	Pathology GCP [14]	Outcome [31]
01	M	60	none	38	>30	VPA, CBZ, TGB	Grade IV	MTS 1B	no GCP	Ia
02	M	44	none	12	5–9	TPM, LVT	Grade III	MTS 1A	no GCP	Ia
03	M	36	none	7	8–10	LVT, PB, CLB	Grade III	MTS 1A	no GCP	Ia
04	F	47	none	33	2–3	PB, CBZ	Grade III	MTS 1A	no GCP	Ic
05	F	33	febrile convulsions	30	5–10	TPM, CBZ, VPA, PB	Grade III	MTS 1A	no GCP	Ia
I	M	55	none	51	10–15	OXC, LTG, CLB	Grade III	MTS 1A	no GCP	Ia
II	F	31	none	16	3–4	CBZ, ZNS	Grade III	MTS 1A	no GCP	Ia
06	F	31	febrile convulsions	10	8–12	PB, TPM	Grade III	MTS 1A	GCP 2	IIa
07	F	33	febrile convulsions	24	3–4	LTG, LVT, PB	Grade III	MTS 1A	GCP 2	Ia
08	F	32	febrile convulsions	27	4–10	CBZ	Grade III	MTS 1A	GCP 2	Ia
09	F	32	febrile conv. (trauma?)	20	9–10	OXC, LVT	Grade IV	MTS 1B	GCP 2	Ia
10	F	37	febrile convulsions	35	12–15	CBZ, TPM	Grade IV	MTS 1B	GCP 2	Ia
III	F	41	febrile convulsions	38	3–4	CBZ, PGB	Grade III	MTS 1A	GCP 2	Ia
IV	M	36	febrile convulsions	22	5–6	TPM, LVT	Grade III	MTS 1A	GCP 2	Ia

CBZ, carbamazepine; CLB, clobazam; LTG, lamotrigine; LVT, levetiracetam; OXC, oxcarbazepine; PB, pentobarbital; TGB, tiagabine; TPM, topiramate; VPA, valproic acid.

### miRNA microarray

**Figure 2:**
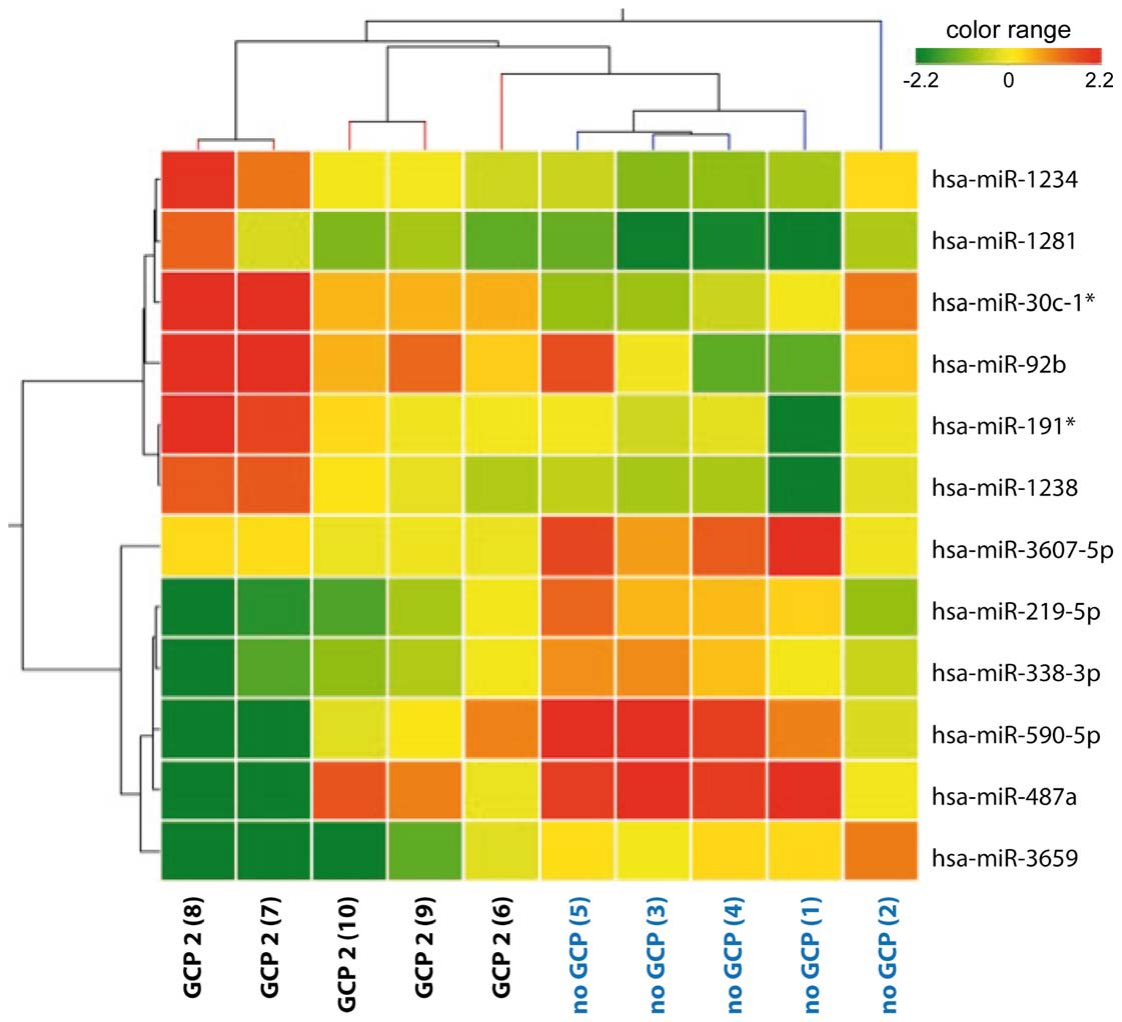
miRNAs differentially expressed in patients without granule cell pathology (no GCP) or with type 2 GCP (GCP 2). Heat-map representation of the average expression of the 12 differentially expressed miRNAs in no GCP and GCP 2 from ten different tissues. The colors of the genes represented on the heat map correspond to the expression values normalized on miRNA mean expression across all samples: green indicates down-regulated; red indicates up-regulated in the tissue. Patients are identified by numbers (in parenthesis) that correspond to those reported in the Table 1.

**Figure 3:**
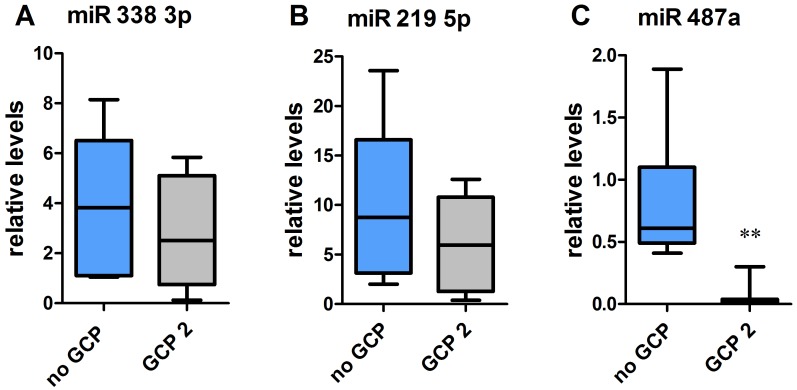
Relative expression of miR-338-3p (A), miR-219-5p (B) and miR-487a (C), evaluated by qRT-PCR in patients without granule cell pathology (no GCP, blue bars) or with type-2 GCP (black bars). Seven patients per group. **P<0.01 Mann-Whitney U test.

Twelve miRNAs were differentially expressed in patients without GCP compared with patients with type 2 GCP (**Fig. 2**). Of these, 6 had relatively higher expression in tissue from patients without GCP and 6 were higher in those with GCP 2 (**Fig. 2**). Differential expression of a subset of 3 miRNAs (namely miR-338-3p, miR-219-5p and miR-487a) was validated in an extended cohort of patients (the ten original patients plus another 2 per group, indicated in roman numbers in **Table 1**) using qRT-PCR. Expression levels of all these miRNAs were apparently different in the two groups, confirming microarray findings, but data were dispersed for miR-338-3p and miR-219-5p, not reaching significance level (**Fig. 3A and 3B**). In contrast, miR-487a expression was confirmed to be highly significantly reduced in GCP 2 (**Fig. 3C**).

### Target identification and validation for miR-487a

Comparative analysis of several databases [16] indicated at least 10 highly likely targets for miR-487a, namely FAM126A, ANTXR1, NUDCD1, AP1S3, AP3D1, AFTPH, KIAA1217, ZNF57 PAG1 and PTGER3. All these mRNAs are expressed in the brain (www.genecards.org). A subset of these are associated with vesicle trafficking (AP1S3, AP3D1, AFTPH), others code for receptors (PTGER3), intracellular signaling (FAM126A) or transcription factors (ZNF57; www.genecards.org). More interestingly, two of these mRNAs (PAG1 and ANTXR1) may be associated with cell adhesion (www.genecards.org). In particular, ANTXR1 (also known as *tumor endothelial marker 8*, TEM8) is expressed in the mouse dentate gyrus granule cell layer (Allen atlas; http://mouse.brain-map.org/gene/show/45380) and has been reported to promote cell spreading in human tumor tissues [20],[21]: therefore, it was hypothesized that reduced expression of miR487a will increase ANTXR1 levels, leading to granule cell spreading (i.e. dispersion or bilamination, i.e. GCP 2).

**Figure 4:**
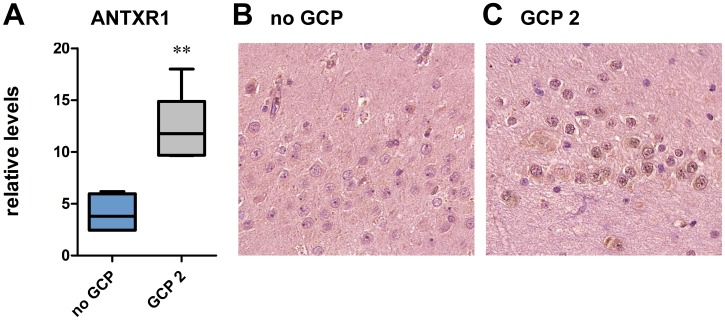
Relative expression of ANTXR1 (A), evaluated by qRT-PCR, in patients without granule cell pathology (no GCP, blue bars) or with type-2 GCP (black bars). Seven patients per group. **P<0.01 Mann-Whitney U test. Representative granule cell layer hippocampal sections from patients without granule cell pathology (B) or with type-2 GCP (C) exhibiting DAB-labeled ANTXR1-like immunoreactivity (LI). Omitting the primary antibody to estimate nonspecific signal yielded completely negative labeling (data not shown). Note a widespread increase in ANTXR1-LI in granule cells from patients with type-2 GCP (C).

Evidence in support of this hypothesis has been pursued by analyzing ANTXR1 mRNA and protein levels in the same samples employed for validation of miR-487a. As predicted, using qRT-PCR ANTXR1 mRNA levels were increased in the GCP 2 group (**Fig. 4A**). Moreover, a clear increase of ANTXR1 immunoreactivity was observed in the granule cell layer of patients with type-2 GCP, as compared with those with no GCP (**Fig. 4B**–**C**).

## Discussion

The main findings of this study were: (1) the identification of 12 miRNAs differentially expressed in the hippocampal granule cell layer of patients with hippocampal sclerosis associated with GCP 2 as compared with patients with no GCP; (2) the RT-qPCR confirmation of one of these, miR-487a, in an extended cohort of patients; (3) the identification of ANTXR1 as a possible target of miR-487a.

An important issue in evaluation of this data is the possible influence of medical treatments on miRNA expression. Indeed, there is evidence that antiepileptic drugs can interfere with miRNA expression: it has been reported that valproate can modulate miR-24, miR-34a, and miR-128 [22] and that phenobarbital can down-regulate miR-122 [23]. Although two patients in the no-GCP group were treated with valproate and five patients (three in the no-GCP group and two in the GCP 2 group) were treated with phenobarbital, none of the above miRNAs was found to be differentially expressed. More in general, a systematic bias due to pharmacological treatments seems unlikely, because all patients in both groups were using many drugs in combination. Therefore, although we cannot rule out an influence of the antiepileptic treatment on miRNAs expression, it seems more likely that the changes we observed are due to the pathology.

The prognosis of patients undergoing epilepsy surgery has been hypothesized to depend on the absence or presence of GCP but, thus far, results have been inconsistent. While some studies reported that GCP does not affect post-surgical outcome [24],[25], others suggested association between GCP and a favorable prognosis [14],[26]. Identification of molecular biomarkers that parallel and/or integrate the pathology findings would provide a valuable prognostic element, and miR-487a may represent a first component of this molecular signature that will allow better patient stratification. In the cohort of patients we analyzed in this study, however, no clear distinction of the outcome was observed in the early timeframe of post-surgical follow-up. Therefore, extension of the follow-up will be needed to verify this possibility.

MiR-487a has been reported to be down-regulated in Alzheimer disease [27] and up-regulated in schizophrenia [28]. Could it play a role in GCP? All miRNAs can have hundreds of targets, but target prediction based bioinformatics approaches is difficult for many reasons, most of all because of imperfect complementarity. However, comparative analysis of several databases [16] indicates at least 10 highly likely targets for miR-487a. ANTXR1 emerged as the most interesting, because it has been reported to promote cell spreading: ANTXR1 is a transmembrane protein that functions as an adhesion molecule, coupling binding of an immobilized extracellular ligand and cell spreading through association to the actin cytoskeleton [20]. Thus, reduced expression of miR-487a could increase ANTXR1 mRNA and protein levels and thereby favor granule cell dispersion. Here, we have provided circumstantial evidence that this could indeed be the case. Further studies in vitro and in animal models are currently ongoing to directly demonstrate this hypothesis.

In conclusion, miR-487a may be the first identified element of a miRNA signature that may be useful for a prognostic evaluation of post-surgical epilepsy and form the basis for mechanistic studies that may lead to the identification of new therapeutic targets, like ANTXR1.
